# Myelin Dynamics at the Axon–Oligodendrocyte Interface: Adaptive Conduction Homeostasis in Demyelination, Remyelination and White Matter Repair

**DOI:** 10.3390/ijms27188280

**Published:** 2026-09-17

**Authors:** Matei Șerban, Corneliu Toader, Răzvan-Adrian Covache-Busuioc

**Affiliations:** 1 Faculty of General Medicine, “Carol Davila” University of Medicine and Pharmacy, 050474 Bucharest, Romania; mateiserban@innbn.com (M.Ș.);; 2 Department of Neurosurgery, “Carol Davila” University of Medicine and Pharmacy, 050474 Bucharest, Romania; 3 Department of Vascular Neurosurgery, National Institute of Neurology and Neurovascular Diseases, 077160 Bucharest, Romania; 4 Puls Med Association, 051885 Bucharest, Romania

**Keywords:** adaptive conduction homeostasis, adaptive myelination, activity-dependent myelination, myelin plasticity, oligodendrocyte mechanotransduction, axon–glia metabolic coupling, oligodendrocyte metabolism, remyelination

## Abstract

In the past, myelination was perceived as a passive, multilayered structure that increased the velocity with which signals travel along an axon. However, evidence now indicates that this conceptualization does not reflect the dynamic processes occurring during the continuous maintenance of signal transmission. Therefore, our hypothesis in this paper is to view myelination as an interactive boundary between the oligodendrocytes enveloping an axon and the axon itself. These cells interact to coordinate several factors, including geometry, membrane structure, nanoscale barriers, cellular accessibility, and metabolic status. For example, TMEM63A mediates Ca^2+^ mechanotransduction in response to mechanical deformation of the sheath, Importin-13 organizes neurofilament localization, and MYO5A supports delivery of Mbp mRNA to sites of local protein synthesis. Additional molecular mechanisms include glycolipid transfer via GLTP for replenishing lipids within the sheath, choline uptake for facilitating sheath lipid renewal, mTOR-mediated regulation of lipid synthesis for supporting sheath composition, and SNAP-23-dependent membrane fusion for providing new membrane components to the sheath. Other mechanisms have been identified to support node and paranode formation necessary for electrical separation. The noncompact regions of the sheath provide additional routes for nonelectrical organelle and cargo movement through the outer-tongue–paranodal-loop–inner-tongue pathway. Lastly, the periaxonal space acts to coordinate ionic, metabolic, and oxidative exchange around the axon. Monocarboxylate transporters are linked to fatty-acid oxidation and lactate-based protein and histone modification such that metabolic flux can be utilized to maintain sheath integrity. We therefore define adaptive conduction homeostasis as the process required to coordinate all of these functions so that the fidelity, velocity, repetitive-firing reliability, and metabolic stability of action-potential propagation are maintained.

## 1. Introduction: Conduction Time as a Living Material State

The speed of the action potential traveling along the length of the axon (the conduction velocity) does not provide a complete picture of a myelinated pathway’s functional capabilities. Other factors also need to be considered. Latency is the time interval prior to the arrival of the first impulse at a particular point after stimulation. Variability in latency among successive impulses (latency variance) is another consideration [1]. Temporal dispersion resulting from convergence of multiple fibers onto a single point affects the pathway’s capacity for accurate timing of impulses. Fidelity of the impulse is an additional concern when considering high rates of impulse generation. Conduction failure due to prolonged periods of activity limits the pathway’s overall functionality. All of the above are related yet distinct from each other. A fiber may have a slower conduction velocity compared with a second fiber and yet maintain consistent timing of impulses [2]. Conversely, a fiber that exhibits reliable spike timing early in an impulse train may demonstrate progressive conduction failure as it continues to generate spikes. Therefore, we define “functional performance” of a fiber as being composed of four separate attributes: “velocity”, “precision”, “reliability”, and “duration” in relation to the electrical transfer of information. Each of these attributes is supported by myelination. Current loss is minimized, effective membrane capacitance is reduced, nodal excitability is maintained, and the spatial organization required for saltatory propagation is preserved [3].

A theoretical basis for understanding the effects of physical properties (diameter of the axon, resistance and capacitance of the membrane, etc.) on signal propagation exists based on cable theory. However, most of these properties are analogously represented as constant parameters. Biological myelinated nerve fibers differ from passive cables in that the majority of components influencing parameter values are continually synthesized, cycled, and metabolically supported. Cytoskeletal organization establishes axonal diameters; lipid synthesis and trafficking create sheath compositions; molecular scaffolds and adhesive complexes at nodes and paranodes influence nodal and paranodal resistance; and continuous transport of proteins, metabolites, RNA, and organelles contributes to maintaining structural integrity [4,5]. Therefore, functional conduction performance is viewed as an emergent property of a sustained axon–oligodendrocyte relationship. The concept of functional conduction performance becomes particularly important as myelination introduces a fundamental biological trade-off. While multilamellar compact membrane architectures restrict radial current flow and minimize capacitive charging, they also effectively separate oligodendrocyte somas from extensive regions of adaxonal membrane requiring continued molecular support [6].

MBP-induced compaction severely reduces cytoplasmic volumes between membrane lamellae; however, CNP-containing noncompact domains provide limited pathways for ongoing molecular movement [7].

Mature sheaths therefore must achieve simultaneous adequacy of closure as electrical insulators and sufficiency of openness as environments permitting membrane renewal, cargo transport, metabolite exchange, redox control, and repair. Furthermore, there is a secondary level of regulation that exists because neither axons nor their sheaths are uniform in spatial distribution. Axonal caliber, local curvature, branching topography, molecular identity, and mechanical load influence internode formation at sites of oligodendrocyte–axon contact. After myelination, the nanoscale organization at nodes and paranodes defines the location and extent of regenerative currents and their confinement. Periaxonal and noncompact domains regulate ionic, metabolic, and molecular exchange. Each interface is dynamic and subject to molecular replacement, organelle relocation, metabolite transfer, and changes in localized redox status based upon activity-related stress or physiological insult [8,9].

Although an internode may appear morphologically unchanged, it may undergo significant alterations in either its electrical competence or its metabolic support. These observations suggests a lack of comprehensive representation regarding myelin structure that cannot be addressed by the commonly employed measures of myelin structure, including g-ratio, sheath thickness, internodal coverage, and/or number of channels at nodes. None of these measures addresses whether the adaxonal surface is accessible, whether the membrane remains appropriately composed, whether the paranodal boundary remains electrically competent, whether cargo arrives at distant locations within the sheath, or whether sufficient metabolic substrates exist to sustain continued activity. Morphologically indistinguishable fibers can thus occupy different functional states; incomplete anatomical restoration can therefore restore meaningful circuit function if the key axons, interfaces, and boundaries responsible for conduction are restored [10].

The biological challenge is therefore not only how much myelin is formed or how much myelin is present, but how a myelinated axon maintains its electrical fidelity and metabolic viability over time. Several defined constructs describe portions of this problem. Myelin plasticity and activity-dependent myelination describe how neural activity, experience, and circuit demand can modify oligodendrocyte-lineage behavior, sheath development, and some aspects of internodal size [11]. Activity-dependent axon–glial communication provides a model describing how neural activity can cause modulation of oligodendrocyte calcium signaling, metabolism, and cellular behavior. Both models provide valuable explanations for how neural activity can modulate myelination or axon–glia interactions [12]. However, the framework proposed here is intended to address a more general query: how is the functional conduction state of an already myelinated axon maintained continuously despite molecular turnover, metabolic demands, mechanical stress, aging, and injury? Not every process that contributes to this maintenance depends upon activity. Examples include lipid replenishment, protein trafficking, nodal–paranodal adhesion, cytoskeletal arrangement, redox management, and substrate utilization for metabolism.

Adaptive conduction homeostasis is a term used herein to describe coordinated regulation of axonal and oligodendroglial processes that maintain conduction performance under diverse physiological and pathological conditions. Velocity is one aspect of a larger functional state that also consists of precision, reliability, and duration in relation to the electrical transfer of information. Homeostasis emphasizes preservation of function in spite of continuous molecular turnover and changing demands on function. Adaptation emphasizes that mechanisms that help maintain this state can be dynamically regulated. Therefore, adaptive conduction homeostasis represents an integrating construct that ties together previous constructs (i.e., myelin plasticity and activity-dependent myelination) with maintenance processes for axonal geometry, sheath material properties, nodal–paranodal boundaries, intracellular access routes to distant interfaces, cargo transport routes, and metabolic support.

In summary, the following sections will examine each stage in sequence: How are axonal geometric relationships established and interpreted when allocating a sheath? How are distinct sections of an axon assigned a specified, compositionally based, and continuously replenished myelin membrane? How are electrically compartmentalized circuits created by nodal–paranodal–periaxonal boundaries from this architectural arrangement? What role does mature myelin play in establishing access to distant interfaces via intracellular transport pathways? Lastly, what roles do substrate utilization rates, lipid turnover rates, organelle position, and metabolic signaling systems play in sustaining the resultant architecture? We will then discuss why failures at different stages in this coupled system result in different paths leading to white-matter dysfunction and why successful remyelination would likely require reconstructing functionally intact interfaces rather than merely increasing membrane coverage.

## 2. Axonal Geometry Is Constructed, Sensed and Topologically Allocated

### 2.1. Importin-13-Dependent Neurofilament Organization Constructs the Calibre Presented to Myelin

The diameters of CNS axons can be quite variable in size. CNS axon diameters can range from approximately 0.1 µm to greater than 10 µm. Also, the diameter of CNS axons can vary independently of axon length, dendritic growth, and neuron body (soma) size. Zebrafish studies involving mutants of the gene ipo13b—an ortholog of the bidirectional nucleocytoplasmic transport receptor importin-13—showed that these mutations disrupted radial growth in Mauthner axons post-embryonically [13]. Mutants of ipo13b had Mauthner axon diameters that were approximately 51% smaller than the corresponding controls seven days post-fertilization. Due to reduced diameter, the cross-sectional area of mutant Mauthner axons was reduced by approximately 80%. Smaller reticulospinal axons were less affected [14].

Mutant zebrafish axons contained significantly fewer neurofilaments than controls. In addition, the remaining neurofilaments had greater densities and shorter nearest-neighbor distances than those in control axons. Both conditions would reduce the degree to which radial expansion could accommodate increased calibre [15]. Neurofilaments are formed from three subunits (NFL, NFM, and NFH) that form polymers. Available space for radial expansion depends on the density and arrangement of neurofilaments as well as their degree of phosphorylation, presence of side arms, degree of cross-linkage, and rate of transport [16].

Importin-13b does not appear to directly regulate neurofilament organization. However, it may transport factors that contribute to the regulation of neurofilament gene transcription, subunit stoichiometry, post-translational modification, polymerization, and/or transport [17,18].

To date, no specific cargo transported by importin-13b has been identified. Thus, these findings represent a nucleocytoplasmic transport–neurofilament pathway whereby neurons control the radial substrate presented to oligodendrocytes for ensheathment. Mutant Mauthner axons were surrounded by myelin sheaths that were similar in thickness to those of control axons. This indicates that radial growth of an axon can occur separately from radial growth of its myelin sheath. Constitutively mutated zebrafish had proportionally fewer spinal-cord axons myelinated than did wild-type animals. Some fibers may not have reached sufficient diameter to permit oligodendrocyte ensheathment [19]. Electrophysiological recordings indicated that increases in conduction velocity during development were more closely related to increases in axonal diameter than to chronological age or apparent sheath thickness. These data suggest that reduced conduction velocity in the absence of alterations in baseline excitability, high-frequency activity, or temporal precision indicates that axonal diameter influences both inclusion in the myelinated population and longitudinal impedance after ensheathment [20].

### 2.2. TMEM63A Converts Wrapping Mechanics into Calcium-Dependent RNA Deployment

TMEM63A-dependent Ca^2+^ signaling provides a possible mechanism for translating mechanical forces into localized synthesis. Mechanically gated ion channels translate physical forces into electrical signals that initiate subsequent biochemical cascades [21]. TMEM63A is structurally characterized as a monomeric channel consisting of eleven transmembrane alpha helices that surround a pore. Structural evidence indicates that TMEM63A interacts with a tightly bound lipid immediately adjacent to its pore. Similarly, the TMEM63A pore environment is influenced by the surrounding bilayer. TMEM63A differs from dimeric OSCA channels located in plant membranes because each TMEM63A monomer appears capable of responding individually to mechanical stimuli [22].

Mechanical input into TMEM63A’s pore will depend on a multitude of mechanisms, including the radius of the axon, local curvature, area contacted by adhesive molecules, available membrane reserve, cortical-actin attachment, and circumferential closure. Increasing membrane tension decreases the free energy required for pore opening. Cholesterol concentration in the bilayer, spontaneous curvature, bilayer thickness, and prestress exerted by the cytoskeleton likely modify the activation threshold. Therefore, TMEM63A can potentially sense total wrapping force instead of functioning as a “molecular ruler” [23].

Deficiencies in TMEM63A in oligodendrocytes led to reduced stretch-induced Ca^2+^ signaling and an uncoupling between axonal diameter and sheath geometry. Sheaths on large-diameter axons were thinner and shorter than those of their calibre-matched counterparts in controls and retracted more frequently. Conversely, sheaths persisted on small-calibre axons that otherwise should have been eliminated [24]. Rather than reflecting a generalized decrease in membrane production, these findings imply that TMEM63A establishes a mechanically defined “window” for determining whether newly formed adhesions are stabilized, extended, or restricted based on local requirements. TMEM63A-initiated Ca^2+^ signaling is involved in linking the detection of mechanical forces to localized RNA delivery. MYO5A and Mbp mRNAs colocalize within nascent sheaths, and loss of TMEM63A leads to reduced MYO5A expression and reduced localization of MYO5A-associated Mbp mRNAs [25]. Hence, TMEM63A initiates a pathway for converting membrane deformation into compaction: Ca^2+^ signaling promotes MYO5A-mediated transport of ribonucleoproteins to sites of localized translation; localized MBP synthesis increases the pool of this highly basic membrane-binding protein; and MBP facilitates dehydration and close apposition of opposing cytoplasmic lamellae while excluding larger cytosolic proteins from compact myelin [26].

### 2.3. Molecular Identity and Axonal Topology Allocate Sheaths Independently of Calibre

Specification of myelin placement is determined by more than simply the diameter of an axon. A neuron’s molecular identity and branching geometry also influence where myelin is placed. Since mechanical cues cannot explain why neighboring axons with the same diameter have different myelination patterns, oligodendrocyte precursors likely utilize molecular recognition systems to identify suitable targets for myelination [11].

Possible candidate recognition proteins include PSD-95 family members, neuroligins, and gephyrin. These proteins reside within punctate structures that transiently appear at defined cellular locations. Sites containing gephyrin overlying areas of localized Ca^2+^ signaling correlate with subsets of stable sheaths [27]. Vesicle release is a significant contributor to Ca^2+^ signaling in OPCs; however, evoked release appears more critical during early stages of development. Disruption of dlg4a, dlg4b, nlgn3b, or gphnb indicates that prospective internodes can be molecularly prepatterned prior to compaction [28].

Gephyrin may acquire greater target specificity after first contacting a target protein. Oligodendroglial gephyrin selectively localizes to sheaths forming around GABAergic and glycinergic axons; loss-of-function mutations in gphnb reduce formation of sheaths on GABAergic axons while increasing the length of those that remain [29].

Competition may arise between target-specific recognition and longitudinal extension along a finite growth program. Gephyrin contains an N-terminal G-domain supporting trimerization and a C-terminal E-domain supporting dimerization. E-domains can self-assemble into filaments promoting liquid–liquid phase separation and clustering of inhibitory receptors in neurons; mutations decreasing this capability (i.e., G375D and D422N) inhibit filament formation. Whether oligodendroglial gephyrin forms an analogous lattice surrounding an uncharacterized receptor, adhesion molecule, or cytoskeletal linker remains unresolved; if so, it could represent adaptation of a conventional postsynaptic scaffold for axonal recognition [30].

In addition to molecular identity, branching geometry imposes further constraints on sheath placement. Live-cell imaging revealed that an oligodendrocyte process can advance beyond an axonal branch point or future node before completely surrounding an axon [31]. Following complete enclosure, the initially continuous contact divides into two compact internodes connected by a thin cytoplasmic bridge. Approximately 31% of oligodendrocytes in zebrafish contained at least one discontinuously wrapped sheath; average lengths for anchor, distal, and bridge segments were approximately 27.4 ± 2.5 µm, 12.4 ± 1.6 µm, and 5.0 ± 0.5 µm, respectively [7].

Each of the 52 Brainbow-labeled cortical bridges analyzed in mice connected internodes produced by the same oligodendrocyte and crossed nodal regions marked by ankyrin-G [32,33]. Ultrastructural electron-microscopy reconstructions showed that each bridge connects the outer tongue of one internode with the outer paranodal loops of the adjacent sheath, creating a cytoplasmic link across nodal regions. Bridges appeared to occur with higher frequency among branched parvalbumin-expressing interneurons: 32.8 ± 3% of their sheaths were bridged compared with 10.8 ± 1% for other oligodendrocytes within the same cortex; approximately 69.1% of bridges intersected branch points; and variation in branch number accounted for approximately 31% of the variation in bridge number [34,35,36]. Table 1 illustrates the hierarchical checkpoints described above, beginning with specification of cytoskeletal architecture in developing neurons and continuing through oligodendrocytic mechanotransduction, molecular target recognition, and branching geometry-dependent determination of individual sheath architecture.

**Figure 1:**
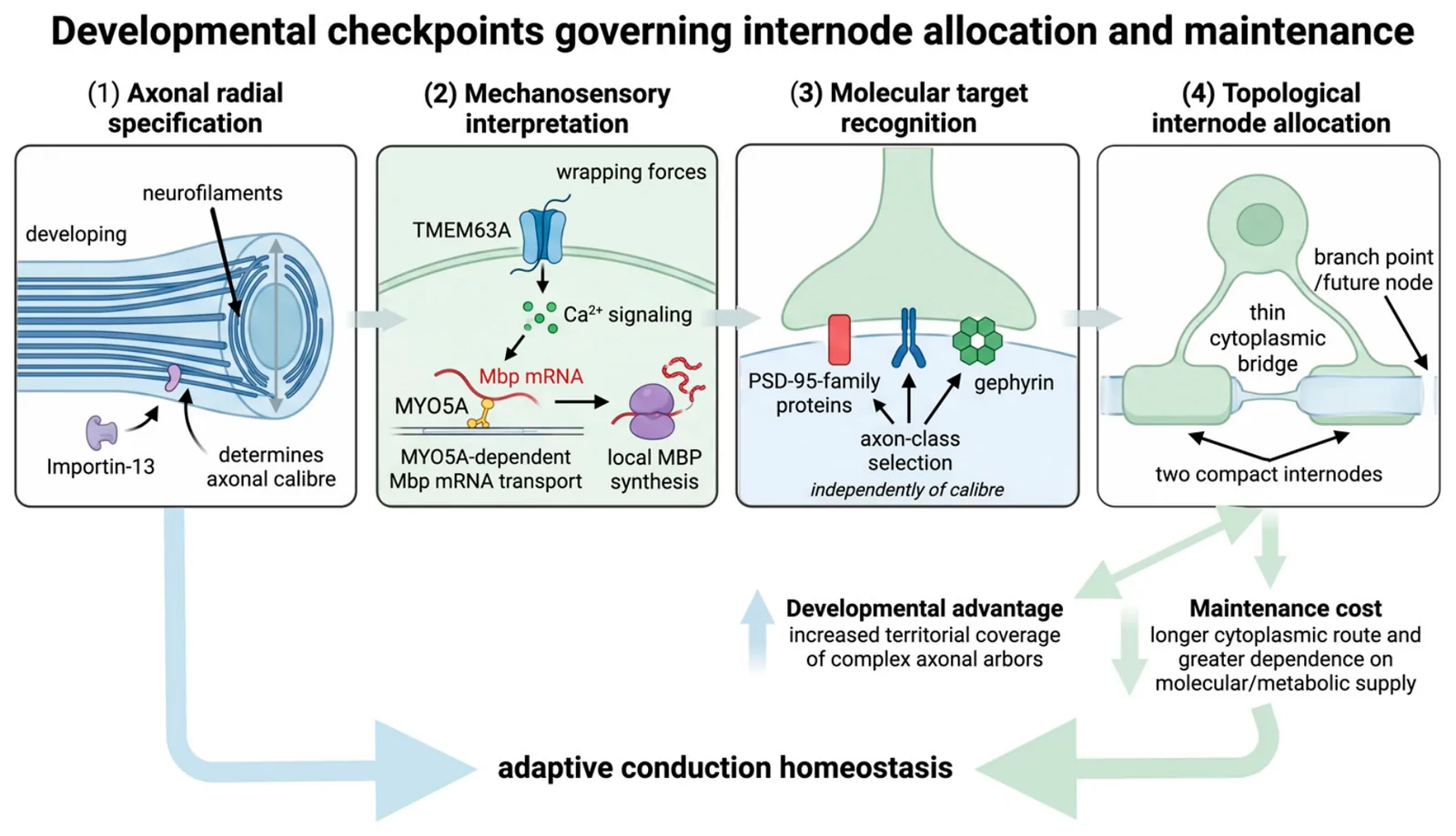
Developmental checkpoints governing internode allocation and maintenance. Axonal calibre is established through Importin-13b-dependent neurofilament organization, mechanically interpreted through TMEM63A-dependent Ca^2+^ signaling and localized Mbp mRNA deployment, and further refined by molecular target recognition and branching topology. These checkpoints determine internode placement and cytoplasmic bridging, balancing developmental territorial expansion against increasing molecular and metabolic maintenance demands within adaptive conduction homeostasis.

Figure 1 depicts these developmental checkpoints leading to the final distribution of internodes along developing arbors and the trade-off between expanding developmental territory and increasing maintenance requirements.

Interconnected sheaths approximately doubled the territory accessible to an individual oligodendrocyte process compared with individual isolated internodes; although this configuration may enhance coverage of complex arborizations during development, it may also establish a longer route for delivery of metabolic and molecular resources [48].

Thus, axonal geometry is specified at multiple spatial scales before mature sheath morphology is completed: radial substrate establishment through importin-13-dependent neurofilament organization; conversion of local mechanical inputs into localized RNA deposition through TMEM63A-dependent Ca^2+^ signaling; and target selection through molecular identity and geometric constraints imposed by branching geometry. These spatial scales establish the basis for subsequent membrane composition, compaction, and long-term maintenance, linking developmental geometry to preservation of adaptive conduction homeostasis [49].

## 3. The Selected Axonal Segment Is Converted into a Compositionally Encoded and Continuously Serviced Dielectric

### 3.1. The Inner Tongue Operates as an Endoplasmic-Reticulum-Coupled Lipid-Transfer Reactor

Oligodendrocytes produce the lipids necessary for the formation of myelin via lipid synthesis. However, they cannot simply move their membrane closer to the axon in order to deposit new layers of myelin. In fact, the distance between the oligodendrocyte and its growing myelin sheath increases significantly during the course of myelination. Therefore, there must be some means of transferring these lipids to the surface of the axon. This process is facilitated by the presence of a continuous tubular network of endoplasmic reticulum (ER) throughout the inner tongue. Specifically, 82% of the myelin-forming processes examined contained ER, compared with less than 20% containing all other types of membrane-bound organelles combined (i.e., mitochondria, Golgi apparatus, etc.) [50]. Furthermore, the ER is extremely close to the myelin sheath. Approximately 62% of all ER profiles were found within 10 nm of the myelin sheath, 83% within 20 nm, and 90% within 30 nm [51,52].

Thus, the inner tongue serves as a site of lipid-transfer reactions linked to the endoplasmic reticulum. Glycolipid transfer protein (GLTP) is present at this interface and is detectable in 92.6 ± 3.1% of oligodendrocytes engaged in active myelination but in only 3.3 ± 1.4% of oligodendrocytes that have not initiated myelination [53]. GLTP functions at this interface by binding glycosphingolipids via an α-helical fold that forms a hydrophobic pocket for incorporation of the ceramide portion of glycosphingolipids. By virtue of this binding mechanism, GLTP allows the transfer of glycosphingolipids between membranes separated by an aqueous space [54]. Galactosylceramide is a likely substrate because it is produced in the endoplasmic reticulum. Whether GLTP interacts with VAP-A at the endoplasmic-reticulum–myelin interface remains uncertain. Likewise, the anchor or tether stabilizing the interaction between myelin and the endoplasmic reticulum remains unclear. Nevertheless, these data collectively indicate that lipid transfer occurs locally at the ER–myelin interface and does not appear to require obligatory transport through the Golgi apparatus [55].

In contrast, deletion of Gltp in oligodendrocytes disrupts both the donor membrane (the endoplasmic reticulum) and the acceptor sheath (the myelin). The inner tongue contains a series of concentric membrane structures derived from the endoplasmic reticulum. Myelin becomes thinner and less organized, with an increased g-ratio and enlarged inner tongues. Additionally, disrupted myelin contains more myelin whorls, lower levels of monohexosylceramides, and altered distributions of phosphatidylethanolamine (PE), phosphatidylserine (PS), and cholesterol [56,57,58,59,60].

It has been suggested that retention of galactosylceramide promotes adhesion and curvature of the endoplasmic-reticulum membrane. While retention limits delivery of galactosylceramide to the myelin sheath, it may generate “rolled” membrane sheets that accumulate as concentric rings within the inner tongue. Therefore, based on these data, we propose that the inner tongue should be viewed as a membrane-contact-site reactor where lipid-transfer reactions occur in conjunction with changes in membrane curvature and adhesion. In turn, these reactions regulate whether newly synthesized lipids become part of the sheath or contribute to aberrant donor-membrane geometry [61,62].

### 3.2. Choline Entry and Sphingolipid Chain Length Specify the Membrane State of Myelin

Rather than relying solely on the absolute quantity of lipids present in myelin, myelin behavior depends on lipid identity and physical state. Conditional knockout of either Slc44a1 or Slc44a5 leads to impaired oligodendrocyte maturation and shortened internodal lengths formed by newly developed oligodendrocytes [63].

**Figure 2:**
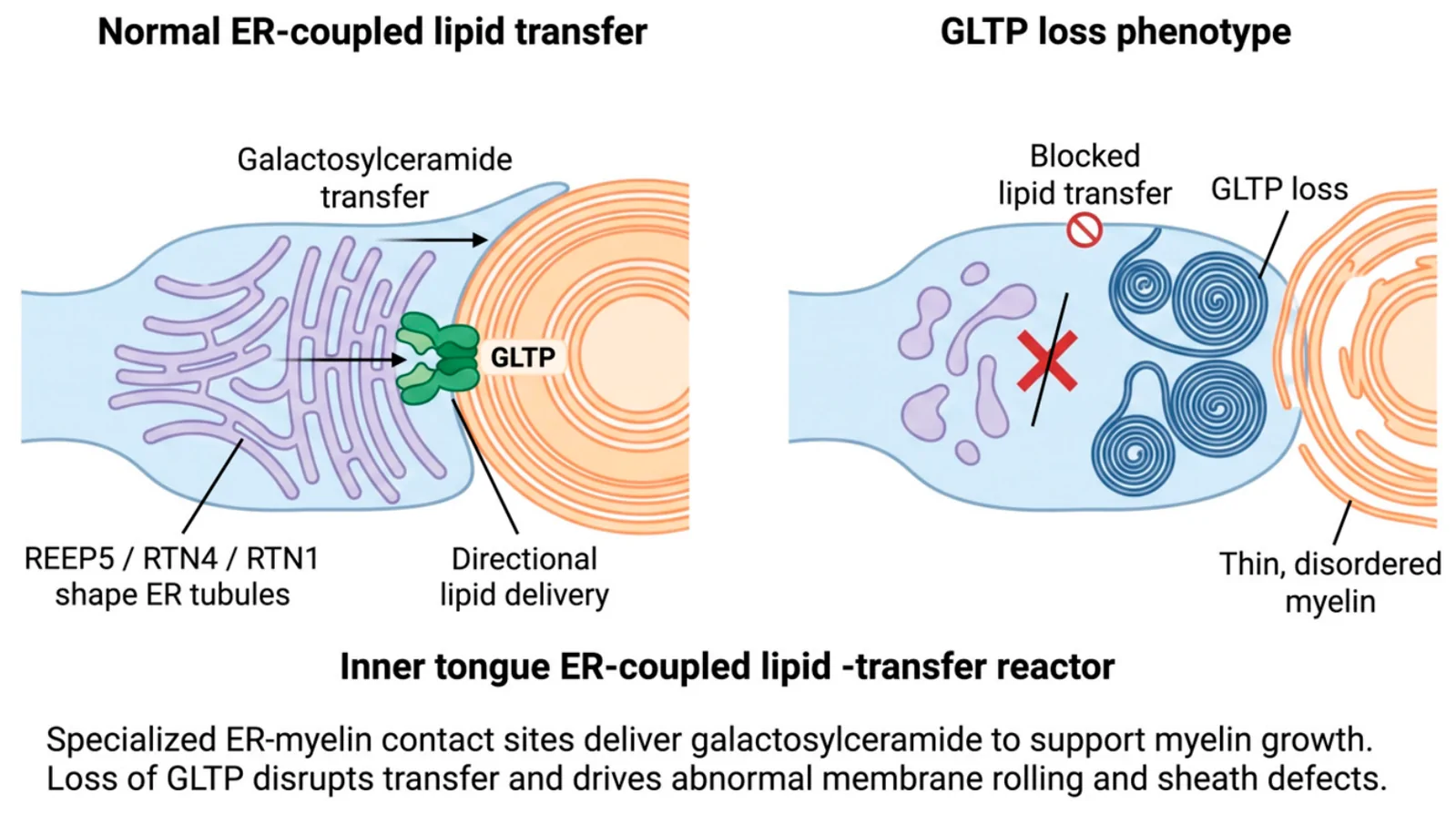
REEP5/RTN4/RTN1-shaped ER tubules form ER–myelin contact sites where GLTP transfers galactosylceramide to the growing inner tongue. GLTP loss causes lipid retention, concentric ER membrane rolling, reduced sheath delivery, and thin, disorganized myelin.

Although Slc44a1 deficiency has more lasting effects on oligodendrocyte maturation than Slc44a5 deficiency, loss of either gene leads to similar impairments in internodal length [63]. In addition to facilitating choline uptake into phospholipid biosynthetic pathways, SLC44A1 also regulates plasmalogen metabolism [64,65]. To illustrate the lipid-transfer architecture described above, Figure 2 shows how the inner tongue functions as an endoplasmic-reticulum-coupled lipid-transfer site.

As indicated above, SLC44A1 deficiency affects the activity of synthetic enzymes involved in phosphatidylcholine biosynthesis. As such, SLC44A1 deficiency leads to reductions in total phospholipid synthesis and alters the overall lipid composition of developing myelin sheaths. Partial restoration of normal developmental progression was achieved using citicoline. Consistent with these findings, biallelic mutations in SLC44A1 have been associated with human leukoencephalopathy [65,66].

Additionally, another mechanism regulating myelin lipid composition is hydrophobic acyl-chain length. For example, ceramide synthase 2 (CerS2) catalyzes the synthesis of C22–C24 ceramides utilized for the synthesis of very-long-chain sphingomyelin, galactosylceramide, and sulfatide [67,68].

Depletion of these lipids caused by CerS2 deficiency results in impaired oligodendrocyte maturation and defective myelination. Furthermore, supplementation with wild-type brain extract restored these deficits in oligodendrocytes grown in vitro. Depletion of very-long-chain sphingolipids has also been shown to disrupt ordered membrane domains and reduce the association of MBP with myelin [69]. Together, these findings demonstrate that headgroup chemistry (lipid species), ether-bonded versus non-ether-bonded lipids, and hydrophobic acyl-chain length provide mechanisms whereby oligodendrocytes regulate membrane order, curvature, and protein distribution within developing myelin sheaths. Although these programs serve similar purposes throughout the nervous system, regional heterogeneity exists. For example, oligodendrocytes obtained from different regions exhibit variable capacities to respond to exogenous lipoproteins [70]. Moreover, regional differences in lipid composition correlate with average axonal diameter. These variations in lipid composition are partly regulated by mTOR-dependent metabolic programming [71].

Therefore, despite possessing similar morphologies, mature myelin sheaths exhibit regional differences in lipid composition, membrane organization, and protein distribution. Regional differences also extend to oxidative stability and energetic requirements [72].

### 3.3. Mature Myelin Is Maintained Through Cargo-Selective Membrane Renewal

For mature myelin to be functional, there needs to be continuous transport of new membrane components made by oligodendrocytes to discrete regions of the myelin sheath. SNAP-23 plays an important role during this process as part of the terminal fusion events. Mice lacking SNAP-23 developed severe motor defects, widespread demyelination, and significant changes in the waveform of compound action potentials (CAPs) along their optic nerves, including slower conduction speed, smaller peak amplitudes, and longer durations before showing significant loss of mature oligodendrocytes [73,74]. Therefore, failure to maintain the myelin sheath can begin long before there is significant loss of the oligodendrocytes responsible for producing it. Three types of evidence converge to show that mature myelin is produced and continually maintained through cargo-selective trafficking, topological specificity, and ultrastructurally organized trafficking:

Failure of Selective Trafficking. Before extensive demyelination occurs, MOG is located adjacent to the nucleus of oligodendrocytes, and later, MAG is found to no longer be localized to its typical position on the abaxonal side [75]. In addition, intracellular MOG colocalizes with giantin but not with EEA1, indicating that the major defect lies within the secretory pathway rather than in the endocytic-recycling pathway. Furthermore, MBP and PLP1 are relatively well preserved throughout this time [76].

Topology of selectivity. MAG and MOG are found at different sides or surfaces of the myelin sheath. As such, failure of a single fusion component can result in selective damage to a particular domain of the membrane while leaving other domains relatively unaffected. Consequently, mature myelin is maintained by cargo- and destination-selective trafficking mechanisms rather than by a nonspecific or general membrane-replacement mechanism [50,77].

Ultrastructural analysis and Ultrastructural Studies. Ultrastructural studies have shown that sheaths derived from mice deficient in SNAP-23 contained numerous large vesicles within their swollen inner tongues, together with axonal spheroids and myelin outgrowths [78]. Recruitment of immune cells occurred after trafficking failure and mislocalization of proteins, indicating a secondary inflammatory response to oligodendroglial membrane-servicing failure. However, no evidence demonstrated that mislocalized MOG acts directly as an antigen [79].

As such, both developing and mature sheaths use complementary maintenance programs. For example, GLTP will serve as the source for glycolipids; SLC44A1 and SLC44A5 will provide lipid precursors; CerS2 will determine the appropriate length of sphingolipid chains; mTOR will regulate lipid composition across different regions; and SNAP-23 will deliver selectively defined transmembrane proteins. Hence, myelin is a continuously serviced insulating material whose function depends not only on the amount of membrane present, but also on lipid composition, molecular structure, and ultimately the type of membrane delivered to specific regions of the myelin sheath [50].

## 4. Nanoscale Boundary Chemistry Converts Sheath Architecture into Saltatory Current

### 4.1. Branched O-Mannose Glycans Delimit the Excitable Membrane

MGAT5B (also known as GnT-IX) is a glycosyltransferase primarily found in the CNS. It plays a major role in forming the glycans necessary for the creation of high-density branched O-mannose structures. These structures contribute to the lateral spacing that defines the boundary between excitable and non-excitable membrane domains. MGAT5B creates high-density branched O-mannose structures by transferring N-acetylglucosamine (GlcNAc) to mannose (Man) through a β1,6 linkage. Neurofascin-186 is a glycoprotein whose extracellular portion forms part of the nodal surface. Its cytoplasmic tail binds ankyrin-G, thus creating a direct connection between the extracellular carbohydrate structure and the membrane domain containing the NaV1.6 channel complex [80,81].

Animals lacking MGAT5B possessed larger nodal areas in multiple cerebral white-matter tracts without decreases in either neurofascin-186 protein or Nfasc mRNA. While the neurofascin-186-positive area at the node did not increase proportionally with the size of the NaV1.6 domain in MGAT5B-deficient animals, its spatial arrangement differed significantly from controls [82]. Thus, MGAT5B-deficient animals failed to properly compartmentalize the nodal organizer laterally, rather than being deficient in the production of its constituent components. Nodal size was normalized by expressing MGAT5B specifically in neurons, indicating a cell-autonomous requirement for MGAT5B in generating the branched glycan [83].

Computational models suggest that hydrated glycan structures generated by O-mannosylation at residues including T203 and potentially T92 lie near interaction sites between neurofascin and contactin-1. The glycan may limit excessive molecular capture or adhesion at these sites, rather than facilitate it [84]. Thus, the glycan may serve as a nodal organizer by limiting excessive molecular association or adhesion [85].

High levels of precision are required to regulate this process. A branched O-mannose structure may allow sufficient molecular spacing and mobility to preserve the boundary between excitable and non-excitable membrane. Additional carbohydrate structures may affect hydration, fixed charge, and macromolecular crowding. Support for this concept comes from studies showing that MGAT5B-deficient callosal axons exhibited increased latency variability after antidromic stimulation and impaired motor performance. Thus, nodal geometry may depend on a glycan-mediated excluded-volume mechanism that regulates adhesion while preserving protein abundance [86].

### 4.2. Mitochondrial Immobilization Converts Local Oxidation into Paranodal Fracture

In order to maintain the structural integrity of the paranode, both axoglial adhesion and continuity of the axonal membrane skeleton must be maintained. On the axonal surface, CASPR1 and its GPI-anchored partner contactin-1 form adhesive complexes across the junction with glial neurofascin-155. The cytoplasmic domain of CASPR1 interacts with protein 4.1B, which links this adhesion complex to βII-spectrin and the subcortical cytoskeleton. As such, these interactions restrict protein movement between nodal and juxtaparanodal domains and minimize current leakage beneath the myelin sheath [87].

Mitochondrial immobilization preceding disruption of this architecture has been shown to occur under conditions of chronic cerebral hypoperfusion. Docking of mitochondria by syntaphilin limits mitochondrial movement and generates local reactive oxygen species prior to obvious demyelination [88]. Oxidizing environments produced by syntaphilin-induced mitochondrial docking have also been associated with abnormal modification of protein 4.1B, retraction of paranodes, and eventual destabilization of myelin. Conversely, syntaphilin depletion restores mitochondrial movement, diminishes oxidative stress in the axon, reduces modification of protein 4.1B, preserves paranodal morphology, and enhances functional recovery [89].

Protection was achieved by modifying mitochondrial positioning rather than increasing junctional proteins or myelin. Stationary mitochondria can produce a localized oxidative microenvironment around vulnerable paranodal adaptor proteins before equilibrium is restored through diffusion and reduction processes. Therefore, mitochondrial residence time at a paranode may represent an important pathological parameter rather than total mitochondrial density or global tissue oxidation [90].

As yet, however, the modified amino-acid residues within protein 4.1B have not been characterized, nor have the effects of these modifications on interactions with CASPR1, spectrin, and membrane components. Available data suggest that biochemical disruption of the junction may predate apparent morphological alterations [91]. Subsequent paranodal displacement can then lead to failure of separation between sodium-channel-rich nodal domains and potassium-channel-rich juxtaparanodal domains (CASPR2/Kv1.1/Kv1.2). Therefore, lesions initiated locally by oxidation of cytoskeletal adaptor proteins can progress from mechanical decoupling and current leakage to dysfunctional excitability, temporal dispersion, and ultimately demyelination [89]. Table 2 presents these mechanisms within a common boundary-chemistry framework, illustrating how glycan-based nodal spacing, paranodal cytoskeletal coupling, and mitochondria-defined redox microdomains control electrical compartmentalization and the fidelity of saltatory conduction.

### 4.3. The Periaxonal Space Is a Return-Current Pathway and Nanoconfined Reaction Compartment

The long, electrically active compartment formed between the internodal axolemma and the adaxonal myelin membrane cannot be considered merely an extracellular gap because it participates in the double-cable model of conduction, in which propagation speed is influenced by current distribution among the axoplasm, periaxonal pathway, and extracellular medium [99]. If the external boundary is highly dissipative, current will be lost more rapidly before reaching subsequent nodes; conversely, if it is more resistive, a larger fraction of current will remain within the fiber-associated circuit, resulting in faster propagation [100].

The effects of axonal diameter, internodal distance, myelin thickness, and nodal size on this circuit depend upon the geometry of adjacent compartments. Therefore, the geometry of the periaxonal pathway dictates both the distribution of return-current paths and the lateral and longitudinal movement of molecules into and out of the compartment [20].

K^+^, H^+^, L-lactate, pyruvate, reactive oxygen species (ROS), membrane fragments, and extracellular proteins may accumulate when their rates of production exceed diffusion through paranodal pathways or uptake across the adaxonal membrane. Confinement of radial and longitudinal flow can reduce electrical current loss but increases the residence time of ions and potentially damaging species [101]. An increase in the width of the periaxonal cleft may improve clearance but can also affect capacitance and current transfer. Therefore, maintenance of functional homeostasis depends upon the relationships among cleft geometry, paranodal permeability, transporter activity, and rates of local production and clearance [102].

Increased repetitive activation results in increased K^+^ efflux, H^+^ production, monocarboxylate exchange, metabolic demand, and oxidative stress. Since proton-coupled transporters respond to both substrate and H^+^ gradients, changes in periaxonal pH can modulate lactate/pyruvate exchange. Slowly cleared ROS and lipid-derived oxidation products can similarly increase the probability of modifying adaxonal proteins without producing detectable changes in bulk-tissue measurements [103].

Proximity labeling directed toward paranodal CASPR1 and internodal MAG revealed local proteomes enriched in proteins involved in cytoskeletal organization, ion-channel organization, septin function, and metabolic regulation. In Alzheimer’s disease tissues, these interfaces showed alterations involving amyloid processing, lipid metabolism, axonal growth, and neuron–oligodendrocyte signaling that were not fully captured in bulk proteomics or single-nucleus RNA sequencing datasets [104].

Expansion microscopy demonstrated amyloid-β fibrils along internodal periaxonal spaces, as well as densely packed coiled deposits near CASPR1-positive paranodes and Kv7.3-positive juxtaparanodes. Amyloid-β deposits correlated with reduced paranodal density, disrupted junctions, and disorganized myelination around dystrophic axons. Despite preservation of gross myelin coverage in certain regions, these findings support an “interface-first” hypothesis in which preserved myelin coverage can coexist with dysfunction of compartments controlling return current, molecular clearance, and axoglial communication [105].

Limited clearance can reinforce local accumulation, inhibit adhesion and transport, and promote structural deterioration that further impairs exchange. White-matter dysfunction may therefore arise from altered molecular spacing, localized oxidation, or restricted clearance despite traditional structural measures indicating intact myelin [106].

## 5. TRAM Converts Noncompact Myelin into an Activity-Coupled Logistics Network

### 5.1. The Outer Tongue, Paranodal Loops and Inner Tongue Form a Continuous Transport Territory

While the cytoplasmic transport channel of the TRAM pathway is geographically discontiguous due to its wide span across internodes and the narrow spaces created by compacting lamellae, the TRAM pathway remains functionally continuous. The TRAM pathway is initiated at the surface of the oligodendrocyte process, continues outward through the outer tongue (OT), proceeds along the paranodal loops (PNLs), and eventually terminates as it enters inward through the inner tongue (IT) and ultimately reaches the adaxonal plasma membrane [24]. Internodes contain two surfaces that meet and converge at each end of the internode. Thus, the TRAM pathway is not simply residual space left behind because of incomplete compaction of myelin sheaths, but rather a folded, continuous space allowing for the movement of organelles from the soma of oligodendrocytes to multiple layers of lamellar sheaths below the adaxonal plasma membrane [50].

Electron microscopy studies performed using thin-section preparations of adult rat optic nerve demonstrate high densities of organelles within IT profiles and in approximately 18% of studied paranodes. Using immunogold-labeled catalase and PMP70 (a peroxisomal membrane protein), ultrastructural studies demonstrated that lysosome-like multivesicular bodies, tubular endoplasmic reticulum, and Golgi-like membrane profiles colocalize in noncompact regions of the same cytoplasmic space. Moreover, localization of Mbp mRNA within mature sheaths demonstrates that TRAM provides not only for organelle distribution but also for ribonucleoprotein access to mature myelin after developmental myelination [107].

Live-cell imaging experiments have shown that organelles such as peroxisomes are actively distributed. Approximately 16% of peroxisomes present in myelin sheaths exhibit movement exceeding 3 µm over ten minutes. Peroxisomal trajectories extending to approximately 44 µm have been recorded. Movement exhibited processive runs interrupted by pauses, reversals of direction, and changes in focal plane [108]. One documented case showed a peroxisome entering what appeared to be an outer tongue, traveling through four successive paranodal loops, and returning toward the oligodendrocyte via what appeared to be an inner tongue. These data support the idea that previously independent compartments can coexist as part of a single transport path [109].

Retention/mobilization of organelles:

Retention/mobilization of organelles likely does not occur randomly. Organelles may be retained at locations where lipid metabolism, oxidative demand, membrane turnover, or local tissue injury occur. After retention, these organelles can be mobilized as a result of internodal condition changes. Mobilization of organelles requires passage through tight channels and abrupt turns where compacted lamellae come into contact with one another without damaging the structure of the sheath. Possible mechanisms for mobilizing organelles involve reversible deformation of organelles, transient expansion of noncompact regions, actin-filament rearrangement, or alteration of motor-protein orientation; however, the actual mechanisms for achieving this remain unknown [110].

### 5.2. Microtubule Polarity, KIF21B and CNP Determine Access to the Adaxonal Membrane

Microtubules organize within oligodendrocyte sheaths and determine the transport of cargo through TRAM. Ultrastructural studies employing thin-section preparations from adult rat optic nerve demonstrated that virtually every oligodendrocyte contains structures positive for β-tubulin IV. Tyrosinated and acetylated populations of tubulin also exist within these structures; therefore, they represent different ages, stabilities, and motility properties associated with different motor proteins [8]. Studies conducted with nocodazole revealed that nearly 40% of oligodendrocytes lost continuity of their β-tubulin IV tracks. Additionally, the distance over which peroxisomal movement occurred decreased from approximately 44 µm to approximately 17 µm. Velocities of mobile organelles underwent little change. Together, these findings suggest that microtubules dictate whether sustained cargo transport can occur and how far cargo can travel [8,111].

The directionality of microtubules within TRAM pathways remains unknown. Bidirectional movement could be due to antiparallel microtubules, although rapid switching between kinesin- and dynein-driven states cannot be discounted. Given that KIF21B transports toward microtubule plus ends and can mediate microtubule polymerization/depolymerization, KIF21B is a prime candidate to regulate both cargo displacement and track formation [112]. KIF21B is concentrated in oligodendroglial white-matter tracts and is elevated in mature oligodendrocyte states under conditions of inflammation/inflammation-derived stress, such as in multiple sclerosis; therefore, KIF21B may be related to increased transport demands derived from inflammatory stress. Conditional deletion of KIF21B specifically within oligodendrocytes led to delayed molecular failure and not immediate loss of myelin [113].

MCT1 levels in myelin-enriched fractions were equivalent to control values in young animals; however, MCT1 levels were significantly reduced in older animals. Similarly, outer-surface MOG and total β-tubulin IV levels were also preserved [114]. This phenotype suggests impaired delivery/incorporation/replacement of specific adaxonal cargo rather than generalized loss of microtubules or compact membrane. Axonal degeneration did not occur until after a prolonged period of molecular failure; this suggests that myelin can remain structurally intact yet progressively fail to sustainably support axonal surfaces [115].

CNP maintains the structural basis for motor-driven transport by preserving open cytoplasmic channels through which microtubules and cargo move. In CNP-deficient myelin, organelles accumulated within enlarged inner tongues before overt neurological defects occurred. Although Slc16a1 mRNA levels were preserved, MCT1 protein levels were significantly reduced, demonstrating a post-transcriptional defect involving trafficking/membrane insertion/protein stability. Similar patterns were seen with physiological aging: larger inner tongues, greater numbers of fibers containing trapped organelles, lower densities of myelinated axons, and approximately 50% fewer MCT1 proteins were found in myelin-enriched fractions [116].

**Figure 3:**
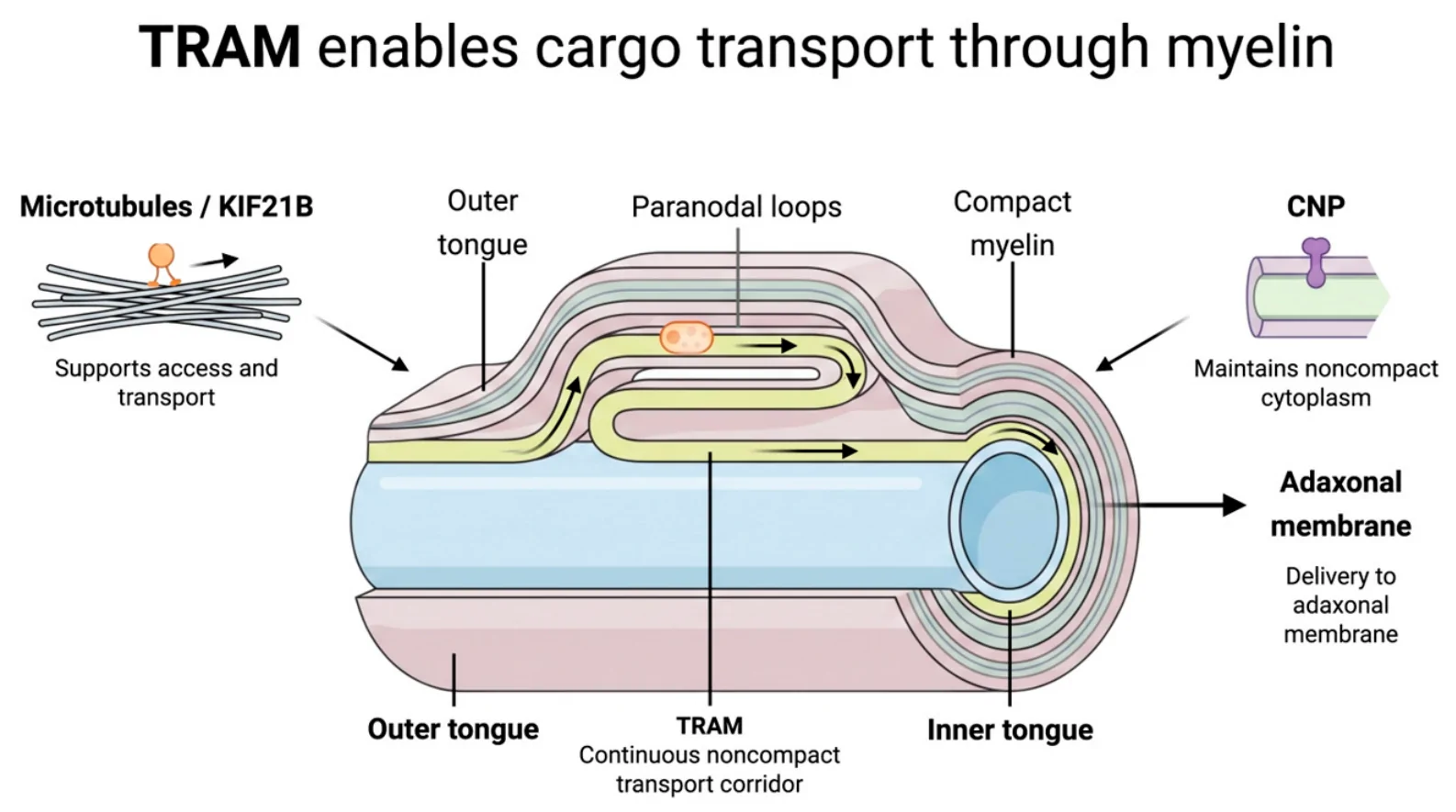
TRAM forms a continuous transport territory extending from the outer tongue through the paranodal loops to the inner tongue and adaxonal membrane. Microtubules and KIF21B support long-range organelle and cargo transport, whereas CNP preserves the noncompact cytoplasmic channels required for trafficking. Together, these mechanisms enable efficient delivery of proteins and organelles to the adaxonal membrane while maintaining functional communication between the oligodendrocyte soma and the mature myelin sheath.

KIF21B and CNP address a common logistical problem by serving different functions. KIF21B directs cargo transport along microtubules and potentially modifies track morphology. Conversely, CNP maintains open channels that allow cargo to traverse long distances toward distantly situated adaxonal domains. Damage to either component would allow compact myelin to preserve structural integrity while functionally isolating axons from surrounding oligodendrocytes [117]. Figure 3 depicts a continuously connected cytoplasmic domain between oligodendrocyte processes and adaxonal membranes, as well as the complementary functions provided by KIF21B-mediated microtubular transport and CNP-mediated preservation of open cytoplasmic channels.

### 5.3. Neuronal Activity Controls Cargo Mobilization Within Individual Internodes

Organelle movement into myelin does not require electrical activity, since peroxisomes enter membranes formed around inert nanofibers. However, once in myelin, movement appears dependent on neuronal activity. Picrotoxin-generated increases in network activity resulted in enhanced peroxisomal movement, while tetrodotoxin-generated reductions in network activity resulted in decreased movement [118]. Such phenomena have also been reported in vivo: awake imaging of the mouse motor cortex found that approximately 20% of peroxisomes moved, while locomotor activity during treadmill running increased this percentage to approximately 30%. Movement typically occurs over tens of minutes within individual sheaths; therefore, activity likely dictates the activation/local operation of motors as opposed to slower alterations in biogenesis/gene expression [119].

Mechanism(s) relating axonal activity to transport:

Repetitive depolarizations produced by axonal activity cause K^+^ release, alterations in H^+^ concentration, glutamate and ATP release, oligodendrocyte Ca^2+^ signaling, increased O_2_ consumption, altered membrane tension, and cellular energy status [21]. Any one or combination of these stimuli could activate motors/adaptor proteins, modify microtubule-associated proteins (MAPs), or alter dimensions of noncompact regions. Glutamatergic/purinergic receptors appear to be potential routes for extracellular stimulation producing intracellular Ca^2+^/kinase signaling. Energy-sensing systems may relate local metabolic requirements to motor recruitment [120].

Reporter organelles:

Given their role in myelin maintenance, peroxisomes are useful reporters. Peroxisomes are involved in VLCFA degradation through acyl-CoA oxidases and/or other enzymes, H_2_O_2_ metabolism through catalase, and ether-lipid biosynthesis. Redistribution may modulate their contributions to these activities. Redistribution will likely not decrease the overall peroxisome population but may alter local rates of each activity mentioned above. Endolysosomal organelles may modulate local rates of membrane/protein turnover, while Golgi-related vesicles may deliver new transporters, cell-adhesion molecules, and enzymes to inner-tongue or adaxonal surfaces [121,122,123].

Data collected so far indicate that cargo movement is dependent on neuronal activity; however, it is not known where specific relocated organelles migrate or what functional implications arise from their relocation. Therefore, peroxisomes should be considered one type of cargo subject to regulation through TRAM rather than the sole functionally relevant cargo [124].

Therefore, TRAM represents a spatially regulated layer bridging electrical activity and maintenance/support of axons. Electrical activity may increase the likelihood that cargo will be mobilized from stationary pools and distributed throughout the sheath toward distant interfaces. Thus, failure of this logistics network may precede overt myelin loss and result in decreased support for axons followed by delayed axonal degeneration [8].

## 6. Metabolic Flux Is Partitioned Between Substrate Capture, Structural Salvage and Covalent State Control

### 6.1. Monocarboxylate Transport Matches Carbon Acquisition to Sheath Demand

Movement of substrates into oligodendrocytes via transporter-mediated net flow across the plasma membrane depends on both the substrate and proton gradients established across the plasma membrane. Consequently, net substrate flow across the plasma membrane will follow the electrochemical gradient prevalent at the time. Movement of lactate, pyruvate, and ketone bodies in either direction is possible based on the local environment. For example, since oligodendrocytes produce lactate through glycolysis, lactate produced in this manner promotes its outward diffusion. Conversely, when glucose availability is reduced and substrate inflow is favored, oligodendrocytes may take up lactate and/or pyruvate [125].

The behavior of MCT2 illustrates this phenomenon. MCT2 is expressed in myelinating oligodendrocytes, and reduced MCT2 expression has been reported in progressive MS lesions. Disruption of MCT2 in white matter of the spinal cord did not lead to a significant loss of oligodendrocyte-lineage cells; however, targeted disruption of MCT2 in oligodendrocytes in the context of inflammation caused by viral delivery resulted in a significant reduction in the ability of those oligodendrocytes to synthesize lipids. Increased expression of inflammatory mediators was also seen, as well as structural damage to the axons; these data are consistent with oligodendrocytes failing to meet their metabolic requirements due to impaired carbon utilization [126].

Impairment of carbon utilization in oligodendrocytes caused by MCT2 disruption resulted in significant axonal injury and demyelination that were reversed by dietary ketosis. The results suggest that carbon sources other than glucose could serve as alternatives for carbon utilization by oligodendrocytes. These data do not support an interpretation limited to impaired lactate efflux from oligodendrocytes. Thus, MCT2 could enable oligodendrocytes to use lactate, pyruvate, or ketone bodies to continue generating acetyl-CoA for oxidative metabolism and fatty-acid synthesis during times of low glucose availability. The high affinity of MCT2 for its substrates may make it uniquely suited to functioning in white-matter environments characterized by variability in metabolite concentrations due to differences in vascular density, diffusion distances, and patterns of neural activation [127].

There remain many questions regarding the physiological functions of MCT2. Some of these include: (1) What are the preferred substrate(s) of MCT2 in oligodendrocytes? (2) What is the distribution of MCT2 in different cellular/membrane domains of myelin? (3) How does inflammation influence the biochemical characteristics of MCT2? We do not think that a single member of the MCT family should be assigned rigid roles in bidirectional substrate transfer. Rather, we believe that each member of this family mediates substrate movement in response to local thermodynamic conditions. The significance of this movement would then be determined by the location of the transport site relative to sites of ATP production, redox balance, and lipid synthesis [128].

### 6.2. Myelin Lipid Turnover Forms an Inducible but Finite Energetic Reserve

Much of the literature characterizes myelin as an energetically costly tissue due to the need for extensive fatty-acid and sterol synthesis for creation of myelin membrane components. However, studies in animal models of white-matter metabolic stress indicate that there is some restitution of this cost through myelin lipid turnover. Degradation of myelin lipids within lysosomes releases fatty acids that can be activated to fatty acyl-CoA for further degradation through peroxisomal β-oxidation or transfer to mitochondria via the carnitine-dependent shuttle system [129]. The resulting acetyl-CoA can be utilized for further oxidation or incorporation into new membrane. Thus, myelin serves as a dielectric and as a pool of lipids that can be recycled back into membrane lost from damaged or aging myelin sheaths; myelin also serves as a conditionally expendable source of carbon for oxidation [130].

This reserve was measured in acutely dissected optic nerves utilizing compound-action-potential recordings in conjunction with genetically encoded measures of axonal ATP. With stimulation at 0.2 Hz and 2.7 mM glucose, conduction was maintained for approximately 2 h after depletion of astrocytic glycogen. Sequential increases in the stimulation rate from 1 Hz to 3 Hz to 7 Hz resulted in sequential reductions in compound-action-potential area, representing progressively worsening propagation failure [131].

Inhibition of fatty-acid oxidation with 25 µM 4-bromocrotonate or 5 µM etomoxir resulted in acceleration of axonal ATP decline and propagation failure. Neither treatment affected conduction at 10 mM glucose, indicating that neither treatment had a functional effect when glucose was plentiful and fatty acids were not required as alternative sources of energy [132].

Peroxisomal and mitochondrial oxidation provided compensation through distinct mechanisms. Complete glucose deprivation resulted in a 70% decrease in white-matter cell viability after 16 h when mitochondrial fatty-acid metabolism was inhibited. Peroxisomal oxidation was less critical for maintaining baseline cell viability. During glucose limitation and repeated stimulation, inhibition of either pathway accelerated the decline in axonal ATP and propagation failure. Optic nerves deficient in oligodendroglial multifunctional protein 2 (MFP2), a key enzyme involved in peroxisomal β-oxidation, demonstrated faster propagation failure during stimulation at 7 Hz [133].

Therefore, it appears that peroxisomes are less important for sustaining basal cell viability than for processing lipid-derived substrates when active axons generate higher and spatially distributed energy demands during high-frequency stimulation. The noncompact regions of myelin place peroxisomes near areas where metabolic support is required; however, how metabolites produced by peroxisomes are delivered directly to the axon has not yet been described [134].

Structural loss ensued when substrate availability declined below the level permitting compensation. Exposing optic nerves to 0.5 mM glucose for 24 h resulted in an increased g-ratio, appearance of submyelinic vesicles, and initiation of oligodendroglial autophagy prior to widespread cell death. Decreases in myelin thickness were similarly seen over time following in vivo oligodendrocyte-specific deletion of GLUT1. Losses in myelin thickness appeared over a period of several months without initial signs of axonal degeneration, gliosis, lipid droplets, or basal conduction deficiencies. This indicates that impaired glucose entry into oligodendrocytes diverted carbon away from membrane production while leaving other metabolic pathways operational [135].

These results strongly support a metabolic-triage model. Under conditions of sufficient glucose availability, oligodendrocytes are able to produce new membranes and maintain their membrane integrity while exchanging glycolytically generated carbon with their respective axons. Once glucose availability drops below a certain threshold, fatty acids created during myelin turnover become directed toward oligodendrocyte oxidation to support cellular function while limiting competition for carbohydrate-derived substrates [136]. Ultimately, under prolonged glucose limitation, degradation outpaces replacement and leads to progressive thinning of the protective sheath facilitating conduction. The membrane protecting conduction is thus used to protect conduction itself. While this process affords temporary protection, the reserve is ultimately limited and used at the expense of the dielectric structure it temporarily preserves [137].

### 6.3. Lactate Translates Carbon Flux into Enzymatic and Chromatin State

Lactate is not simply a product being moved across the plasma membrane; production of lactate is tightly linked to cytosolic redox regeneration through lactate dehydrogenase A (LDHA). When LDHA generates lactate, it simultaneously reduces pyruvate and oxidizes NADH to NAD^+^, thereby replenishing the necessary cofactors for continued glycolysis. Diminished glycolytic output and lactate production have been documented in demyelinated lesions, as well as a general decrease in lysine lactylation. These relationships between glycolytic activity and oligodendrocyte function may arise from one or both mechanisms: insufficient substrate and removal of a metabolically responsive protein modification [138].

Administration of lactate or overexpression of LDHA in oligodendrocytes enhances myelin regeneration in models of experimental demyelination, whereas lineage-specific deletion of Ldha impairs developmentally dependent myelination and causes neuropathy. Proteomic analysis as well as lactylation analysis indicated that modification influences the activities of LDHA and carbonic anhydrase II, among others; these modifications promote additional lactate production, suggesting a positive-feedback mechanism whereby glycolytic flux promotes the enzyme responsible for supporting glycolytic flux [139]. Similarly, lactylation promotes oligodendrocyte maturation; however, consequences of lactylation on zinc-dependent hydration of CO_2_, proton transfer, and regulation of intracellular pH remain undefined [140].

Modification of existing enzymes involved in metabolism through non-histone lactylation permits rapid adjustments in glycolytic/acid–base functions without necessitating transcriptional changes. Histone lactylation modifies chromatin state/accessibility over longer time scales than enzyme modification because it influences transcriptional accessibility. Elevated histone H3 lysine 27 lactylation was correlated with increased Olig2 expression, oligodendrocyte-lineage progression, and myelin regeneration in a rat spinal-cord-injury model. Therefore, lactate may regulate metabolic support through rapid enzymatic and chromatin-based alterations [141].

Special attention must be given when evaluating the chemical basis underlying metabolic pathways that involve lactate. The major acyl donor for oligodendroglial lysine lactylation has not been defined; hence, enzymatic transfer using lactyl-CoA must be differentiated from nonenzymatic modification by reactive lactoyl intermediates. Also unknown are modification stoichiometry, site specificity, enzymatic turnover rates, and potential structural effects on LDHA oligomerization or carbonic-anhydrase catalysis. As such, increased lactylation cannot be assumed to always yield positive outcomes; rather, the outcome will most likely be dictated by the type(s) of residue(s) modified, proteins modified, state(s) of cells undergoing modification, and duration of exposure [142].

Regardless of remaining uncertainties, lactate provides a clear link between metabolic flux and cell state. Availability and usefulness of carbon are regulated by monocarboxylate transporters, access to usable energy from stored structural lipids is mediated by lipid oxidation, and conversion of metabolic history into enzymatically accessible and non-histone protein-modifying information is mediated by lactylation. Overall, these processes define how axon–oligodendrocyte units establish and sustain conduction under varying states of demand, as well as how prolonged compensation results in maintenance of electrical properties while continually modifying the molecular and material composition of white matter [143].

## 7. White-Matter Failure Occupies a Path-Dependent Conduction-State Landscape

### 7.1. Conduction Competence Is a Distributed Molecular State Rather than a Structural Endpoint

The characteristics of the conduction of an axon depend on the degree of myelination, and the status of all of the above-mentioned components (the axonal cytoskeleton, the node of Ranvier scaffold, the paranodal adhesion complex, the periaxonal return-current path, the axoglial transport apparatus and the oligodendrocyte metabolic activity) are interdependent. As a consequence, it is theoretically possible for myelin to remain morphologically intact and yet for the proteins involved in nodal function to be improperly expressed (such as neurofascin, for example, and paranodal connections), thus impeding the ability of the transport apparatus of noncompact myelin to move properly [144,145].

The study of cortical remyelination provides evidence for this. Thyromimetics administered in high doses restored normal visual-response latencies in animals whose oligodendrocytes and myelin accounted for about 89% and 97% of those found in control animals, respectively. Lower-doses of thyromimetics produced oligodendrocytes and myelin levels of about 87% and 96% of those found in controls, but did not produce an improvement in visual-response latency. Moreover, vehicle-treated cortex, which underwent remyelination after lesioning, showed no increase in visual-response latency [66,146].

The results of these studies provide clear evidence that the mere presence of a significant amount of myelin is not sufficient to restore the functional properties of conduction. In addition to the issue of the total quantity of myelin being too low, there are numerous reasons that recovery may occur without correlation with total myelin quantity. To begin with, axons may not be repaired optimally, internodes may not develop correctly, the mechanical properties of the myelin sheath may still be immature, the nodes of Ranvier may not be geometrically uniform, paranodal seals may not form completely, and/or there may be insufficient ionic/metabolic support for repetitive firing [50]. Other factors that might reduce substrate availability for repair include increased energy consumption during repetitive activity, paranodal leakage, and/or reduced efficiency of lipid recycling [147,148,149].

When morphology appears relatively stable, a single nerve fiber can exist in a compensated state and may ultimately succumb to further activity or additional insult beyond its capacity to generate ATP, transport cargo, and replenish molecules [150].

### 7.2. Disease Trajectories Enter the Landscape Through Distinct Molecular Coordinates

Depending upon the entry point of a demyelinating disease into the conduction-state space, considerable axonal loss can occur as it travels throughout that region. Recently, a group used single-cell RNA-seq and chromatin sequencing data from over 150k oligodendrocytes to determine rapid inflammatory activation and induction of antigen-presentation pathways [151]. Months after transcription had ceased, loci related to immune processes remained open to modification, thereby demonstrating long-lasting molecular memory and potential influence upon thresholds or responses to subsequent insults [152,153].

In addition to inflammatory activation, oligodendrocyte subtype-specific responses were also identified. MOL2 oligodendrocytes demonstrated elevated immune programming, while MOL5 and MOL6 oligodendrocytes exhibited increased accessibility of regenerative pathways at later time points. Thus, inflammation produces several distinct states showing varying degrees of immunogenicity, metabolic activity, stress response and regenerative capacity [128,154].

Other issues relevant to initial conditions in white-matter regions include:

Regions within white-matter contain differing levels of oligodendrocyte density, lineage dynamics, myelin content, axonal size distribution, internodal architecture and metabolic programs. Therefore, similar types of damage can elicit very disparate responses among different white-matter regions or developmental stages [155].

After chronic demyelination and chronic axonal injury along with chronic glial activation and lymphocytic invasion following repeated injury to Tmem10 positive oligodendrocytes with resultant spontaneous remyelination and precursor-cell proliferation, incomplete repair can persist between successive injury events [156].

As a result of both prior state of the system and manner in which that state was achieved, disease severity will vary among individuals [157].

Autoimmune injury initially affects chromatin and cellular identity, vascular insufficiency subsequently reduces mitochondrial redox stability at the paranodes, aging increases limitations to cytoplasmic access and impairs transport, and amyloid pathology leads to pathological accumulation in the periaxonal space. Although these convergent disease pathways culminate in demyelination as a common endpoint for disease progression, therapeutic targets preceding demyelination may be uniquely linked to each disease [158].

### 7.3. Repair Is Constitutive, Thresholded and Molecularly Non-Stoichiometric

Adult OPCs continuously attempt differentiation at variable spatiotemporal rates independent of demand; however, these attempts decrease with age and acute inflammation. It would seem that successful remyelination requires coincident occurrence between injuries and lineages progressing towards differentiation [159].

Repair involves equilibrium among three processes: oligodendrocyte production, interface degeneration and axonal tolerable limits. Slow development of injury allows sufficient time for internode replacement before metabolic/oxidative stresses become severe enough to kill axons. Rapid development of injury exposes axons to prolonged metabolic/oxidative stress until adequate internode replacement can occur. Regardless of the rate of injury development, new myelin develops within damaged tissue characterized by altered nodal boundaries, extracellular matrix and metabolic environment [160].

Repair is molecularly non-stoichiometric. Equivalent amounts of membrane newly synthesized during recovery can exhibit markedly different lipid compositions, internodal lengths, alignment/targeting with axons, configurations of channels domains, and/or formation of paranodal junctions [161]. Therefore, functionally immature membranes may remain even though they represent substantial quantities relative to control levels, while smaller quantities of strategically located mature myelin may facilitate timing pathway establishment before complete structural normalization [162].

Both new oligodendrocytes and their contacted axons play critical roles in successful recovery. Aberrant programs expressed by oligodendrogliocytes can “lock-in” as a result of inflammation. Axons themselves may undergo alterations in cytoskeletal structure, organelle distributions, and surface organization, thus forming new myelin on a substrate that is not neutral, and restoring coverage does not equate to restoring original molecular organization [66]. Consequently, functional recovery should be assessed using measures such as conduction latency, variability, and fidelity during repetitive stimulation as well as axonal integrity and maturation of nodal, paranodal, and adaxonal organizations [48].

Effects from therapeutic agents may also be nonlinear: circuit timing may improve once a minimum number of fibers reach maturation thresholds, while widespread remyelination fails to restore function if metabolic coupling, transporter replacement, or axonal viability remains limiting [163].

Ultimately, success depends on whether therapy effectively shifts the system past its relevant failure boundary in the conduction-state space versus merely increasing tissue quantity [164].

## 8. Conclusions

There are numerous structural aspects of myelin that increase the rate of signal transmission by increasing transverse membrane resistance and reducing membrane capacitance. In addition to speeding signal transmission, myelin acts as a physical barrier to the movement of molecules throughout the internode. Therefore, the effectiveness of myelin depends upon a number of geometric characteristics of the internode, such as the dimensions and locations of the nodes. The effectiveness of myelin also depends upon dynamic characteristics of the internode, including the rates at which lipids and proteins are exchanged locally. Additionally, specific structural characteristics, including nodal and paranodal regions, control the flow of ions and provide noncompact pathways for molecular exchange. Together, these seemingly conflicting design requirements define a primary problem in myelin biology: compacted membranes reduce both capacitance and current leakage; however, they also limit diffusion of replacement proteins, organelles, and metabolites into and out of distantly located portions of the membrane. Therefore, compactness and accessibility represent opposing design constraints that must continually be reconciled within the same biological system.

Myelin represents a multilevel collection of operational systems. At the first level, the biochemical characteristics of the sheath are established through acyl-chain selection during fatty-acid synthesis, phospholipid synthesis, and fusion of cargo vesicles with the plasma membrane. Glycosylation, axoglial adhesive interactions, and cytoskeletal attachment organize these biochemical properties into electrically insulating domains. Microtubule-based transport systems then provide distant biochemical constituents throughout the sheath. Lastly, monocarboxylate exchange, lipid metabolism, and stimulus-dependent post-translational modification of proteins regulate conduction and metabolic support. Since each of these systems functions at a distinct yet interconnected level, compensation within one system cannot necessarily overcome failure of another.

Therefore, studies examining white-matter disease should take into account pathology beyond simple loss of membrane. White-matter fibers may remain structurally sheathed while failing to accurately position their nodes, seal their paranodal regions properly, facilitate adequate axonal transport, clear materials from the periaxonal space, and/or maintain sufficient metabolic support. Before demyelination becomes apparent by microscopic examination, some or all of these deficits may lead to increased latency variability, temporal dispersion of action-potential propagation, synchronization errors, and decreased fidelity of repetitive action-potential discharge.

Furthermore, structural repair of demyelinated white matter does not guarantee functional recovery. Functional recovery is contingent upon accurate axonal targeting, restoration of internodal geometry, proper placement of nodes relative to internodes, sealing of the paranodal region, appropriate lipid composition, and maintenance of cytoplasmic access and metabolic exchange. Consequently, structural endpoints should be evaluated simultaneously with measures of conduction velocity, latency variability, impulse dispersion, repetitive-firing fidelity, and molecular indicators of mature nodal, paranodal, and adaxonal organization.

Adaptive conduction homeostasis defines a conceptual basis for integration of these additional factors. By continually adjusting properties commonly viewed as static, conduction velocity, timing precision, and endurance can be maintained. White-matter disease is therefore path-dependent. Inflammation alters cellular state; vascular stress damages redox-sensitive junctions; aging decreases transport and renewal capabilities; and protein aggregates damage nanoscale interfaces. Therefore, histologically similar lesions may result from completely different molecular trajectories with entirely different limiting steps.

Significant gaps in our knowledge remain. These include identification of cargo responsible for linking nucleocytoplasmic transport to neurofilament organization; determination of how mechanically gated Ca^2+^ signaling regulates localized mRNA trafficking/translation; resolution of the directionality of microtubules crossing myelin sheaths; and definition of concentration gradients within the periaxonal space. Most, if not all, of these mechanisms have been examined only indirectly in human white matter. Significant progress will depend upon the development of techniques that retain spatial and temporal relationships among biological processes, including interface-restricted proteomics, cryostructural imaging, long-term organelle tracking, genetically encoded metabolic sensors, and concurrent electrophysiology.

Thus, the central issue has transitioned from “what” is in myelin to “where” these elements move, “how quickly” they are replaced, “which” exchanges become limiting, and “when” compensatory mechanisms cease to function.

A vast body of evidence supports a surprisingly simple conclusion: myelin does not rely upon structural integrity alone for its maintenance but requires continuous molecular activity. Lipids are transported, proteins undergo turnover, organelles are relocated, metabolites cross membrane boundaries, and nanoscale interfaces are continually stabilized to sustain conduction.

The central inquiry thus transitions from “How does an axon obtain insulation?” to “How does a compacted membrane continue to function?”

Repair of white-matter disease therefore must restore more than a layer of membrane—it must rebuild boundaries capable of sustaining conduction velocity, precision, and endurance.

## Figures and Tables

**Table 1:** Quantitative structural measurements integrated with the molecular mechanisms governing axonal calibre specification, mechanosensitive Ca^2+^ signaling, localized RNA deployment, axon-class recognition, and topological internode allocation, illustrating how geometric information is progressively translated into stable myelin organization across multiple spatial scales.

Developmental Checkpoint	Principal Molecular Regulators	High-Resolution Structural Features	Functional Consequence	Experimental Signature	References
Radial calibre specification	IPO13, NEFL, NEFM, NEFH, phospho-NFH/NFM, kinesin/dynein transport	Axons 0.1–>10 μm; ipo13b KO ~51% diameter reduction (~80% CSA); reduced NF number, increased filament packing, shorter nearest-neighbour spacing	Neurofilament abundance and spacing determine radial expansion, axial resistance and conduction substrate	Cell-autonomous ipo13b mutants, TEM morphometry, single-axon electrophysiology	[34]
Cytoskeletal mechanical template	Neurofilament side arms, spectrin-actin lattice, microtubules, plectin, ankyrins	NF phosphorylation controls side-arm extension; interfibrillar spacing defines cytoskeletal volume; radial lattice stiffness scales with filament organization	Establishes mechanical geometry presented to oligodendrocytes before wrapping	Cryo-ET, filament-density mapping, phosphoproteomics	[37,38]
Mechanosensory thresholding	TMEM63A, membrane cholesterol, cortical actin, phosphoinositides, Ca^2+^	Monomeric 11-TM mechanosensitive channel; gating determined by membrane tension, curvature, adhesion area and cortical prestress	Converts integrated wrapping mechanics into localized Ca^2+^ microdomains	Cryo-EM, membrane stretch assays, Ca^2+^ imaging	[39]
Local biosynthetic commitment	Ca^2+^, MYO5A, MBP mRNA, RNP granules, actin tracks	TMEM63A loss reduces MYO5A abundance and MBP transcript localization; local translation precedes membrane compaction	Mechanical input is translated into polarized MBP synthesis and internode growth	smFISH, live RNA imaging, translational reporters	[25,40]
Axon identity recognition	PSD95, Neuroligin-3, Gephyrin, MAGUK complexes, vesicular glutamate/GABA signalling	Stable gephyrin-positive puncta with local Ca^2+^ transients prefigure future internodes	Molecular identity biases sheath stabilization independently of diameter	OPC-specific mutants, live imaging	[41]
Sheath allocation logic	Gephyrin G/E domains, Z-interface polymers, inhibitory adhesion complexes	G375D/D422N disrupt higher-order assembly; preferential localization on GABAergic and glycinergic axons	Axon-class recognition competes with longitudinal sheath extension	Super-resolution microscopy, lineage-specific deletion	[42]
Topological internode distribution	Outer tongue continuity, paranodal loops, Ankyrin-G, branch architecture	31% bridged oligodendrocytes; anchor 27.4 ± 2.5 μm; bridge 5.0 ± 0.5 μm; distal segment 12.4 ± 1.6 μm	Single processes distribute cytoplasm across multiple internodes and branch points	Volume EM, Brainbow reconstruction	[43,44]
Development-maintenance trade-off	Cytoplasmic bridges, metabolic transport, ageing-associated degeneration	52/52 bridges connected sister internodes; 69.1% crossed branches; branch number explained ~31% of bridge variance; bridged sheaths ~10× more degeneration-prone	Maximizes developmental territory coverage while increasing long-term vulnerability	Longitudinal ageing imaging, serial EM	[45,46,47]

**Table 2:** Breakdown of how MGAT5B-dependent O-mannose branching, neurofascin–contactin partitioning, CASPR1-NF155-protein 4.1B coupling, and syntaphilin-regulated mitochondrial redox microdomains cooperate to preserve nodal geometry, paranodal sealing, sodium-potassium channel segregation, and conduction fidelity, and how disruption of these mechanisms promotes current leakage, temporal dispersion, and progressive demyelination.

Nanoscale Boundary Module	Principal Molecular Architecture	Biophysical Mechanism	Structural Consequence	Electrophysiological Impact	Representative Evidence	References
Glycan-defined nodal boundary	MGAT5B (GnT-IX), branched O-mannose glycans, NF186	β1,6-GlcNAc branching generates hydrated steric exclusion and fixed-charge microdomains	Sharp confinement of NF186 and NaV1.6 nodal territory	Maintains compact excitable membrane and low conduction jitter	Neuron-specific Mgat5b deletion/rescue	[80]
Neurofascin partitioning	NF186, ankyrin-G, βIV-spectrin, actin lattice	Extracellular glycan architecture coordinates extracellular spacing with intracellular anchoring	Stable nodal membrane geometry despite unchanged NF186 abundance	Preserves NaV clustering and nodal stability	Super-resolution imaging; biochemical analysis	[92]
Adhesion buffering	NF186, CNTN1, O-mannosylated Thr203/Thr92	Branched glycans sterically limit neurofascin–contactin association rather than maximizing affinity	Prevents excessive molecular capture and lateral domain expansion	Optimizes nodal precision instead of adhesive strength	Structural modelling; co-immunoprecipitation	[93]
Paranodal mechanical coupling	CASPR1, CNTN1, NF155, protein 4.1B, βII-spectrin	Trans-cellular adhesion mechanically couples terminal loops to the axonal membrane skeleton	Preserves paranodal seal and molecular diffusion barrier	Restricts current escape and ion-channel mixing	Cryo-EM, ultrastructure, knockout studies	[94]
Redox-sensitive adaptor stability	Protein 4.1B, ROS, syntaphilin-docked mitochondria	Local ROS oxidize load-bearing cytoskeletal adaptors before global oxidative injury develops	Progressive weakening of CASPR1-spectrin linkage	Early paranodal instability preceding demyelination	Oxidation assays; chronic hypoperfusion models	[95]
Mitochondrial spatial signaling	Syntaphilin, axonal mitochondria, antioxidant systems	Mitochondrial dwell time determines cumulative local oxidant exposure	Organelle positioning regulates nanoscale redox microdomains	Spatial rather than global oxidative burden predicts junction failure	Live mitochondrial tracking; syntaphilin knockdown	[96]
Domain segregation failure	Protein 4.1B, CASPR1, Kv1.1, Kv1.2, CASPR2, NaV1.6	Mechanical uncoupling permits lateral membrane diffusion across paranodes	Paranodal retraction and erosion of nodal boundaries	Current leakage, temporal dispersion and reduced conduction fidelity	Electrophysiology; immunolabeling; morphometry	[97]
Saltatory conduction integrity	Glycan architecture, paranodal adhesion, cortical cytoskeleton, mitochondrial positioning	Coordinated extracellular, membrane and cytoskeletal organization maintains nanoscale electrical insulation	Stable node-paranode geometry despite physiological stress	High conduction velocity, low latency variability and preserved motor coordination	Multimodal structural and functional analyses	[98]

## Data Availability

No new data were created or analyzed in this study. Data sharing is not applicable to this article.
